# amica: an interactive and user-friendly web-platform for the analysis of proteomics data

**DOI:** 10.1186/s12864-022-09058-7

**Published:** 2022-12-09

**Authors:** Sebastian Didusch, Moritz Madern, Markus Hartl, Manuela Baccarini

**Affiliations:** 1 Max Perutz Labs, Vienna Biocenter Campus (VBC), Dr.-Bohr-Gasse 9, 1030 Vienna, Austria; 2 Vienna BioCenter PhD Program, Doctoral School of the University of Vienna and Medical University of Vienna, Vienna, Austria; 3 University of Vienna, Center for Molecular Biology, Department of Microbiology, Immunobiology and Genetics, Dr.-Bohr-Gasse 9, 1030 Vienna, Austria; 4 University of Vienna, Center for Molecular Biology, Department of Biochemistry and Cell Biology, Dr.-Bohr-Gasse 9, 1030 Vienna, Austria

**Keywords:** Proteomics, LC-MS/MS, Web application, Data analysis, Data visualization

## Abstract

**Background:**

Quantitative proteomics has become an increasingly prominent tool in the study of life sciences. A substantial hurdle for many biologists are, however, the intricacies involved in the associated high throughput data analysis.

**Results:**

In order to facilitate this task for users with limited background knowledge, we have developed amica, a freely available open-source web-based software that accepts proteomic input files from different sources. amica provides quality control, differential expression, biological network and over-representation analysis on the basis of minimal user input. Scientists can use amica’s query interface interactively to compare multiple conditions and rapidly identify enriched or depleted proteins. They can visualize their results using customized output graphics, and ultimately export the results in a tab-separated format that can be shared with collaborators. The code for the application, input data and documentation can be accessed online at https://github.com/tbaccata/amica and is also incorporated in the web application.

**Conclusions:**

The strong emphasis on dynamic user interactions, the integration of various databases and the option to download processed data, facilitate the analysis of complex proteomic data for both first-time users and experienced bioinformaticians. A freely available version of amica is available at https://bioapps.maxperutzlabs.ac.at/app/amica.

**Supplementary Information:**

The online version contains supplementary material available at 10.1186/s12864-022-09058-7.

## Background

Mass spectrometry (MS)-based proteomics enables deep qualitative and quantitative characterization of any organism’s proteome which is crucial for the understanding of the underlying cell biology, physiology and biochemistry. The constant technological advancement of instruments and data-acquisition techniques, as well as the parallel development of a broad variety of methods such as proximity-dependent labeling for the study of weak or transient protein-protein interactions (PPIs), has expanded the scope and relevance of MS-based approaches in tackling specific biological questions. As a result, proteomic approaches have become increasingly popular, but the complex analysis of MS-based proteomics data is an obstacle for many novices in the field, complicating and delaying the interpretation of experimental outcomes.

Moreover, the many software platforms available for processing proteomics raw data and their different output formats require advanced knowledge for obtaining final interpretable results. Several attempts to solve this problem have been made in the past. The software Perseus [1], for example, is widely used because it provides a graphical user interface and has extensive options for the analysis of both label-based and label-free methods. Other software tools, for example MSstats [2] or MSnbase [3] revolve around the R programming language. These tools have the advantage of automating many of the data processing steps that would have to be performed manually in the Perseus interface, but require knowledge of the programming language R.

Recently developed applications integrate their software into R-Shiny apps, allowing for interactive user interactions and visualizations of the output from the MaxQuant package, one of the most widely used software platforms in MS-based proteomics [4]. LFQ-Analyst [5] enables the automatic analysis of label-free data, Eatomics [6] allows for the input of enhanced experimental designs and ProVision [7] can process label-free and TMT labeled data and integrates PPI networks. These tools provide appealing solutions for first-time users but are limited to output from MaxQuant. While Protigy (available at https://github.com/broadinstitute/protigy) permits the upload of generic user input, none of these tools allow the systematic comparison of proteins across multiple groups or the integration and comparison of multiple proteomics experiments.

As a solution to these issues, we have developed amica, a user-friendly web-based platform for comprehensive quantitative proteomics data analysis that can automatically handle multiple database search tool outputs such as for instance data from FragPipe - a recent but increasingly popular open-source software package [8, 9] - as well as any generic tab-separated dataset such as RNA-seq data and datasets that were previously analyzed with amica. amica’s built-in query interface facilitates the identification of molecular entities (proteins or RNAs) specific to biological groups. The output graphics allow immediate visualization of qualitative and quantitative differences among groups. Finally, omics data obtained by different methods (e.g. RNA-seq and mass spectrometry) can be directly compared, allowing multi-omics integration.

### Comparison with other tools

A comparison of amica with currently available R-Shiny apps for proteomic data analysis (LFQ-Analyst, ProVision, Eatomics and Protigy) is shown in Table 1, and underscores amica’s versatility.

**Table 1:** Comparison with other tools

	LFQ-Analyst	ProVision	Eatomics	Protigy	amica
Generic file input				x	x
Reuploadable output				x	x
Query interface					x
Biological networks		x		x	x
Dot plots					x
Reports	x		x	x	x
Customizable output graphics	(x)	x	(x)	(x)	x
Available at public webserver	x	x	x		x

### Implementation

amica is implemented as an open-source, interactive R-Shiny app that provides generic user input, quality control and differential abundance analysis for quantitative proteomics data.

amica uses established software tools and libraries and integrates various biological databases. The front page of amica and Table 1.1 in the supplementary material lists all references that should be cited, when using amica to analyze a data set and to generate output graphics.

#### Upload: accepted input file formats

amica can read in common database search tool output formats, custom formats and its own tab-separated format. It is able to achieve this by mapping file specific column names into common features present in proteomics data. These include a unique protein id, a gene name, different type of processed intensities, peptide counts, spectral counts and other common columns in proteomics output formats. A description of column names from analyzed data mapped to amica’s format is shown in Table 2.

**Table 2:** amica format. Column names of amica’s output format. All intensities are log^2^ transformed

Column name	Description
Majority.protein.IDs	Unique Protein ID
Gene.names	Gene names
spectraCount	Total MS MS count
razorUniqueCount	Total Razor + unique peptides
razorUniqueCount.<sample>	Razor + unique peptides per sample
iBAQ_<sample>	iBAQ intensity per sample
RawIntensity_<sample>	Raw Intensity per sample
LFQIntensity_<sample>	LFQ Intensity per sample
ImputedIntensity_<sample>	Normalized and imputed intensity per sample
Potential.contaminant	Potential contaminants have a “+” in this column
*P*.Value_	*p*-value for a pairwise group comparison, e.g *P*.Value_group1__vs__group2
adj.*P*.Val_	adj. *p*-value for a pairwise group comparison, e.g adj.*P*.Val_group1__vs__group2
logFC_	log2 fold change for a pairwise group comparison, e.g logFC_group1__vs__group2
AveExpr_	Avg. intensity for a pairwise group comparison, e.g AveExpr_group1__vs__group2
quantified	Filtered proteins contain a “+” in this column

Three different data upload options are available in amica (see Additional file 1: Fig. 2): a) a database search result such as MaxQuant’s proteinGroups.txt file (see Additional file 1: Table 2.1), FragPipe’s combined_proteins.txt file (see Additional file 1: Table 2.2), b) a custom tab-separated file or c) a previously analyzed data set in amica’s format. All three options require a tab-separated file denoting the experimental design in the experiment (see Additional file 1: Table 2.4), i.e. the mapping of samples to distinct biological groups. For option a) and b) a tab-separated file containing a contrast matrix that specifies the desired pairwise group comparisons to be made needs to be uploaded (see Additional file 1: Table 2.5). Finally, for custom file uploads in option b) an additional specification file, that maps common column features in proteomics output formats to amica’s format needs to be uploaded (see Additional file 1: Table 2.6).

#### Analysis options

Users can filter proteins on the basis of minimum count values for MS/MS counts and razor + unique peptides. Additionally, proteins can be filtered by valid values per group, e.g only proteins identified in a minimal number of replicates are kept for analysis (see Additional file 1: Fig. 3). Sample intensities (e.g raw intensities or LFQ intensities) can be selected for log$_2$ transformation and normalization. Normalization options include no normalization, quantile normalization, variance stabilization normalization (VSN) and median normalization. Two different methods for differential expression analysis can be selected, limma [10] and DEqMS [11]. Finally, three different imputation methods for missing values can be chosen: i) lowest detected value, ii) randomized sampling from a normal distribution with a user specified downshift and width for each sample or iii) globally for all samples.

#### Example data

The example data was taken from an interaction protemics study [12]. This includes raw files for four groups from an interaction proteomics study focusing on PGRMC1, a protein from the membrane-associated progesterone receptor family with a variety of cellular functions. In this study, MIA PaCa-2 cells were stably transfected with a PGRMC1-HA plasmid and Co-IPs of PGRMC1 interacting proteins were isolated from cells expressing PGRMC1-HA, as well as from non-transfected parental MIA PaCa-2 cells as a negative control, with and without AG-205 treatment (a PGRMC1-specific inhibitor).

#### Data processing

MaxQuant (version 1.6.17.0) was used to analyze the raw files. As search database, UniProt UP000005640 (downloaded on 10th September 2021) was used, with Trypsin/P as proteolytic enzyme allowing for two missed cleavages. The match between runs (MBR) feature was not used. Oxidation on methionine and protein N-terminal acetylation were set as variable modifications, and Carbamidomethylation of Cysteine was set as fixed modification. Label-free quantification and normalization was performed with the MaxLFQ algorithm [13].

Additionally, FragPipe (version 16) with MSFragger [14] (version 4.0.0) and Philosopher [9] (version 4.0.0) was used to analyze the raw files. Label-free quantification and normalization was performed with the MaxLFQ algorithm by IonQuant [15] (version 1.7.5). The same search database as well as variable and fixed modifications as for MaxQuant were used for FragPipe. Peptide validation was executed by Percolator [16] (version 3.05). The MBR feature was not used.

The output from MaxQuant and FragPipe were further analyzed using the same analysis parameters in amica. Briefly, proteins with at least 2 Razor + unique peptides, at least 3 MS/MS counts, and valid values in 3 out of 5 replicates in at least one group were considered quantified. LFQ intensities of quantified proteins were log2-transformed and missing values were imputed from a normal distribution downshifted 1.8 standard deviations from the mean with a width of 0.3 standard deviations. Differential expression analysis was performed with limma. Both sets of results can be downloaded from the example datasets in amica. Only the results of the MaxQuant analysis are shown here.

## Results

**Fig. 1:**
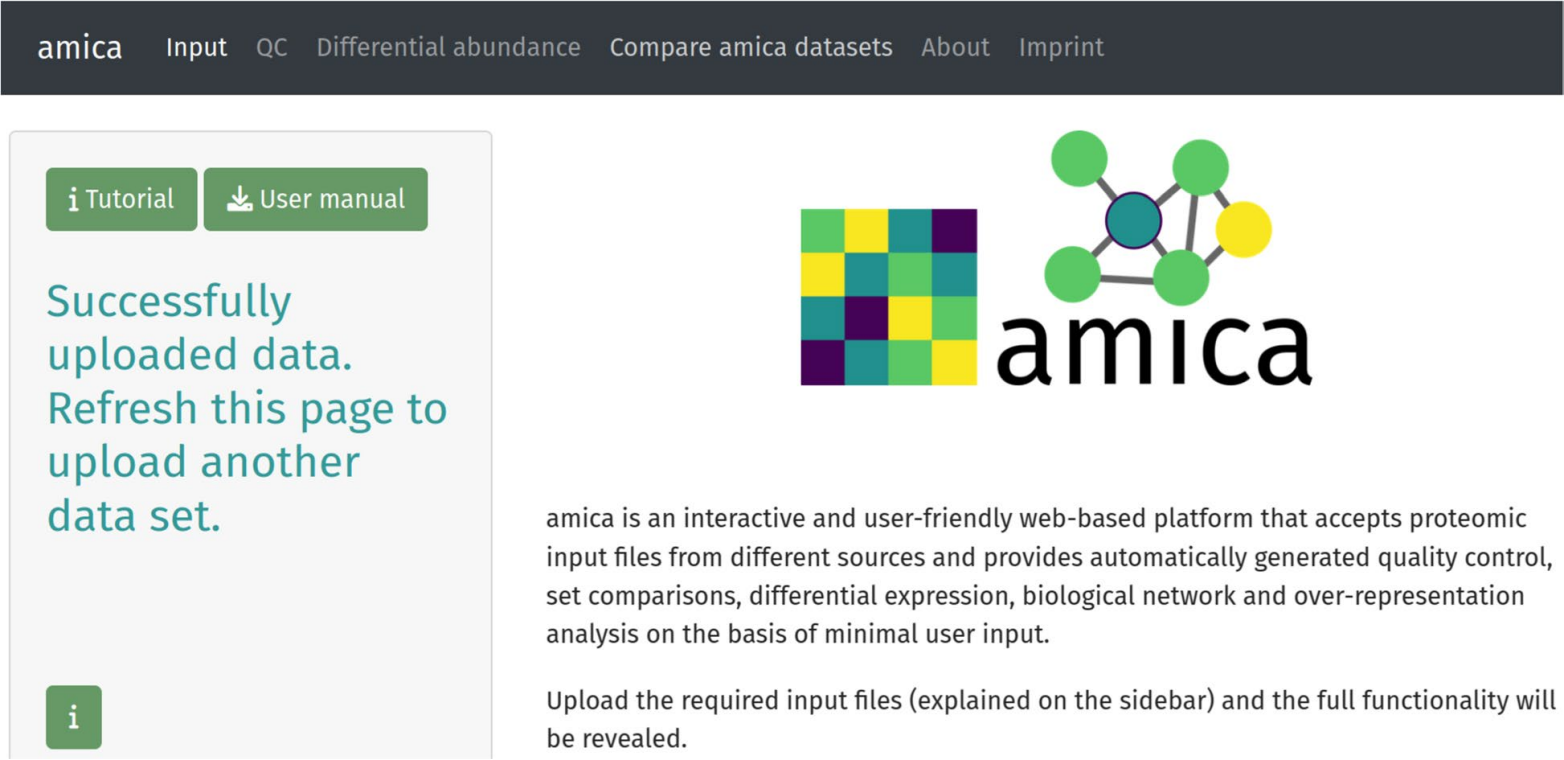
A screenshot of amica’s user interface visible after uploading the input files

amica is developed as a user-friendly web application with interactive and customizable visualizations that can be exported in a publication-ready vector graphic format.

amica’s landing page displays online documentation, a link to a user manual and a link to download the example data set in all allowed input formats (Fig. 1). The landing page also serves the purpose of the user input tab. After successfully uploading the required input files, amica generates a downloadable file in its custom format. This file can be used as input file for subsequent re-inspection, analysis and visualization in amica (see Additional file 1: Fig. 4).

In the input tab, users can also define the colors to be used for visualizations (see Additional file 1: Fig. 5). Color palettes can be either chosen from ColorBrewer [17] or defined by a color picker tool. This includes i) qualitative colors to label biological groups of the experimental design, ii) qualitative colors for various types of scatter plots and iii) color gradients used for heatmaps and correlation plots. Once chosen, colors propagate through all visualizations in amica, allowing for coherent output graphics. The input tab allows the users to define a specific order in which the biological groups will be displayed on the plots axes and legends. This feature proves especially useful for visualizing time series data.

In the Quality Control (QC) tab, users can generate and inspect plots at different processing levels of the data, such as raw intensities, LFQ intensities, iBAQ intensities and normalized and imputed intensities. This makes it easy to examine the impact of common pre-processing steps (normalization and imputation). An automatically generated report containing user selected analysis parameters, and plots comparing intensities before and after normalization and imputation can be downloaded, allowing for reproducible output and analysis.

**Fig. 2:**
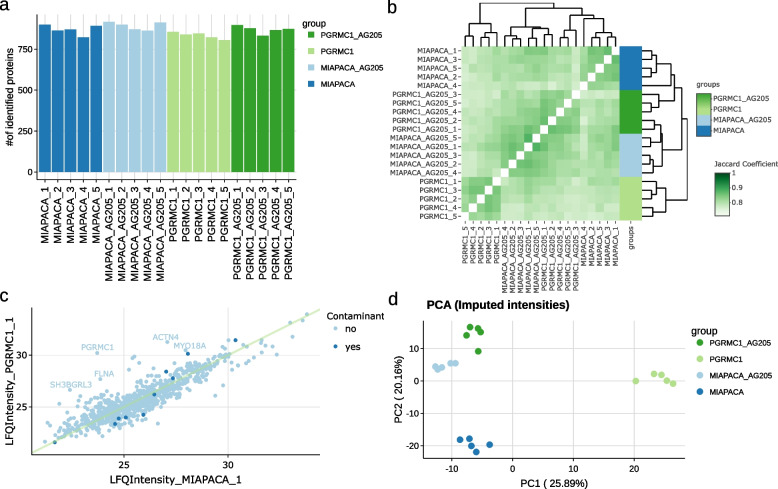
Examples of amica graphics, generated using the provided example dataset. **a** The number of identified proteins can be visualized in a bar plot. **b** The overlap of identified proteins between samples can be shown in interactive heatmaps such as the Jaccard coefficient shown here. Other visualization possibilities are the numbers of shared proteins or the overlap coefficients (not shown). **c** Scatter plots can be generated using different types of intensities and samples. Users can interactively highlight proteins and choose to draw a straight line, a linear regression line or no line in the scatter plot. **d** A PCA plot can help in identifying clusters

The available visualizations for the intensity distributions in density - and box plots for different samples are particularly useful in this respect (see Additional file 1: Fig. 6). A barplot for the number of identified proteins (Fig. 2a), a sample overlap heatmap of identified proteins (Fig. 2b), as well as a scatter plot (Fig. 2c) and a boxplot of coefficient of variations (see Additional file 1: Fig. 7a) make it possible, to evaluate the reproducibility of replicates. A correlation plot (see Additional file 1: Fig. 8b) and a principle component analysis (PCA) plot (Fig. 2d) allow to identify clusters in an exploratory data analysis in the QC tab. Last but not least, when iBAQ intensities are available, barplots for the percentage of missing values (see Additional file 1: Fig. 9b), the percentage of intensity of the most abundant proteins, and the percentage of intensity of potential contaminants per sample allow for the detection of outliers (see Additional file 1: Fig. 11).

The Differential Abundance tab is the heart of amica, enabling the analysis and visualization of quantitative proteomics data. As a first step, users can specify common thresholds to subset protein groups of interest, such as log$_2$ fold change thresholds, thresholds on *p*-value or adjusted *p*-value, and select only enriched, only reduced, or all differentially abundant proteins in pairwise group comparisons. In the next step, users can select single or multiple pairwise group comparisons to which these thresholds should be applied. An automatically generated differential abandance report summarizing the results can be downloaded.

**Fig. 3:**
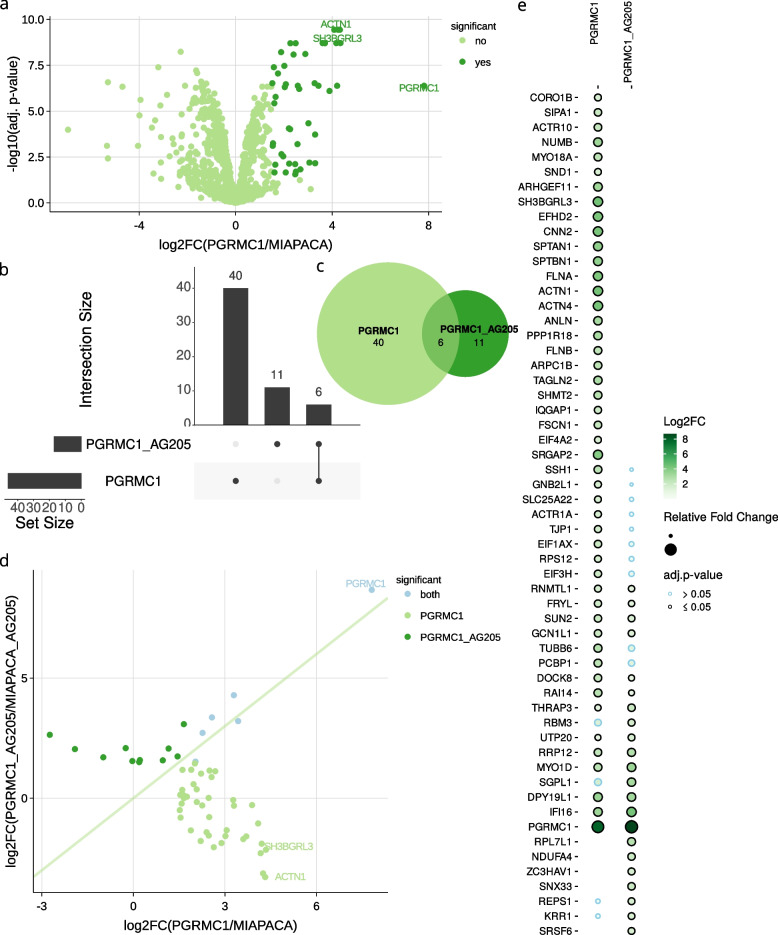
**a** Differentially abundant proteins can be represented with volcano plots for a single group comparison. Upset plots (**b**) and Euler diagrams (**c**) can be generated to visualize different sets of proteins from multiple comparisons. Fold-change plots (**d**) or dot plots (**e**) can be created to visualize multiple comparisons quantitatively

Differentially abundant proteins can be visualized as volcano (Fig. 3a) - and MA - plots (see Additional file 1: Fig. 12b) for single group comparisons. Unlike other R-Shiny apps for proteomics data analysis, amica can generate UpSet plots [18] (Fig. 3b) and Euler diagrams (Fig. 3c) for visualizing the overlap of significant proteins from multiple selected group comparisons. Differentially abundant proteins are displayed in a data table that can be exported as a csv file for further analysis (see Additional file 1: Fig. 14). The columns of the data table show the gene names of proteins, statistical information such as log$_2$ fold changes, *p*-values, adjusted *p*-values, and a binary sign (yes or no) of significance for all user-selected comparisons. In addition to the filters applicable to each column, users can use the gene name column to further subset the data table by specifying search patterns using regular expressions. amica’s unique query interface allows to immediately inspect differentially abundant proteins that are part of a specific process or pathway (see Additional file 1: Section 7.2).

In order to compare two conditions quantitatively, amica can generate a scatterplot of log$_2$-fold changes on the x-axis and the y-axis (fold-change plots; Fig. 3d). Significant changes are highlighted in color. amica integrates dot plots for the visualization of quantitative and statistical information of more than two conditions (Fig. 3e). In this type of plot, every row corresponds to a protein, and every column to a pairwise group comparison. Proteins are displayed as circles, whose sizes and colors can be either selected as intensity or log$_2$ fold change. The line color of the circle shows the statistical significance of a protein in a group comparison.

**Fig. 4:**
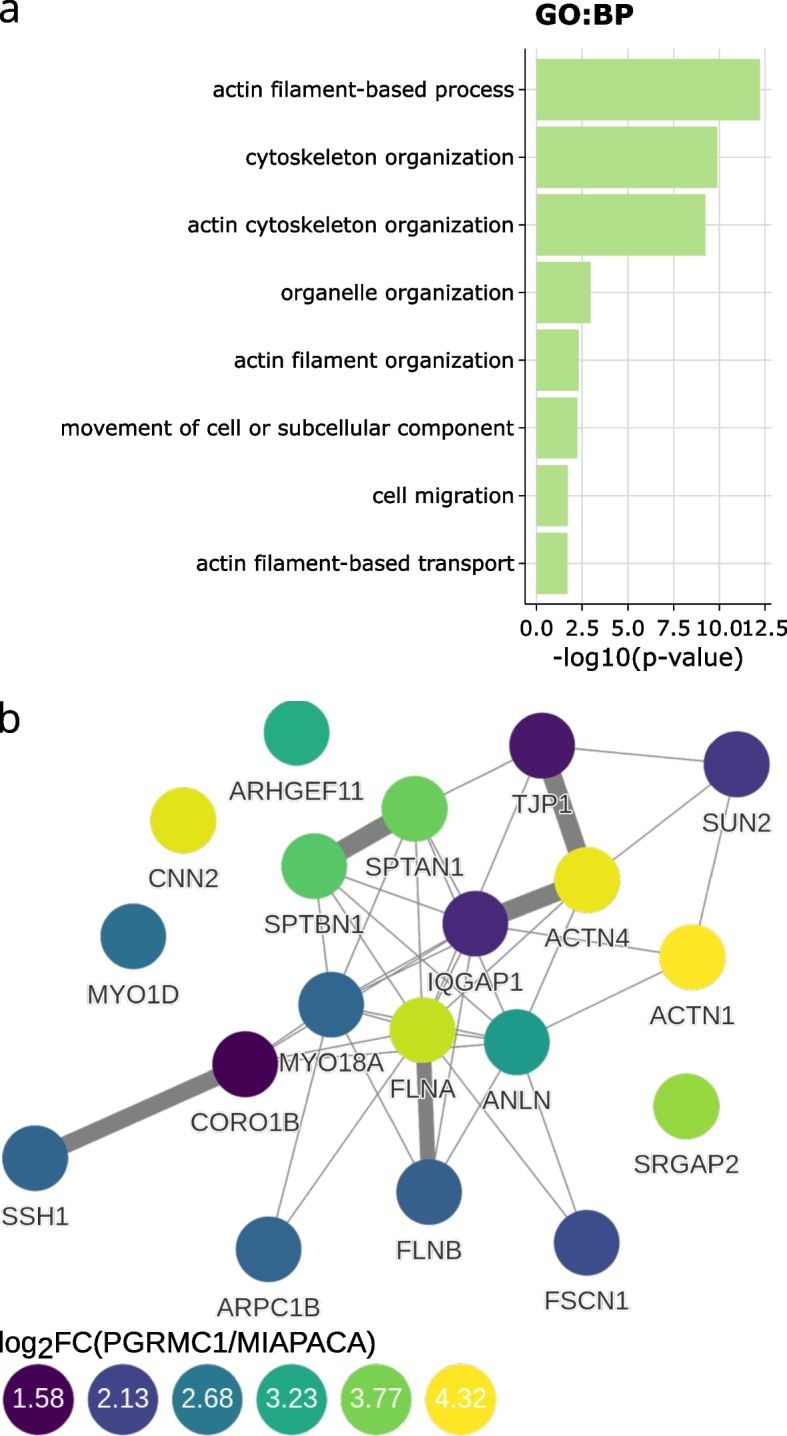
**a** Over-represented biological processes resulting from a functional enrichment analysis performed using gprofiler2. **b** Differentially abundant proteins annotated with the most singnificant term “actin filament-based process” are integrated in a PPI-Network from the IntAct database. Log$_2$ fold changes are displayed as a color gradient of the nodes

In addition to visualizing proteins across multiple groups, interactive heatmaps [19] (see Additional file 1: Fig. 15) can be created to compare and cluster many proteins, or profile plots can be created for a single protein (see Additional file 1: Fig. 20).

For human proteomics studies, amica allows users to map proteins to a PPI network from the IntAct database [20] (Fig. 4b), and enables the retrieval of subcellular localization predictions from the humancellmap database [21]. As an example, selecting a particular cellular compartment in the interactive web interface will highlight all proteins that map to this localization. This information can be downloaded in gml format for visualization in a network visualization tool such as Cytoscape [22] (a tutorial on visualizing amica’s output in Cytoscape is described in Additional file 1: Section 7.3).

A functional enrichment analysis of differentially abundant proteins using gprofiler2 [23] assists in building hypotheses on the underlying biology. Users can select the organism of the experiment and its corresponding data sources (see Additional file 1: Fig. 21). The output of gprofiler2 is shown as a manhattan plot (see Additional file 1: Fig. 22a) and as a barplot (Fig. 4a) for a user selected data source. Additionally, a downloadable output table with all data sources, term names, *p*-values and corresponding gene names is displayed (see Additional file 1: Fig. 23). The gene names from this table can be used to construct a regular expression to subset the query data table, allowing for the visualization of proteins belonging to a specific gene set.

Finally, the “Compare amica datasets” tab gives the possibility to upload previously analyzed datasets to be compared with the current data input (see Additional file 1: Fig. 24). As a key column, the two datasets can be combined by protein ID or gene name. After successfully uploading a second amica file, a scatter plot and a correlation plot becomes available for correlation analysis of the combined dataset (see Additional file 1: Fig. 25). In addition, a selection box will appear at the top of the Differential Abundance tab, allowing users to use amica’s query interface on the integrated datasets (see Additional file 1: Fig. 26).

## Conclusions

**Fig. 5:**
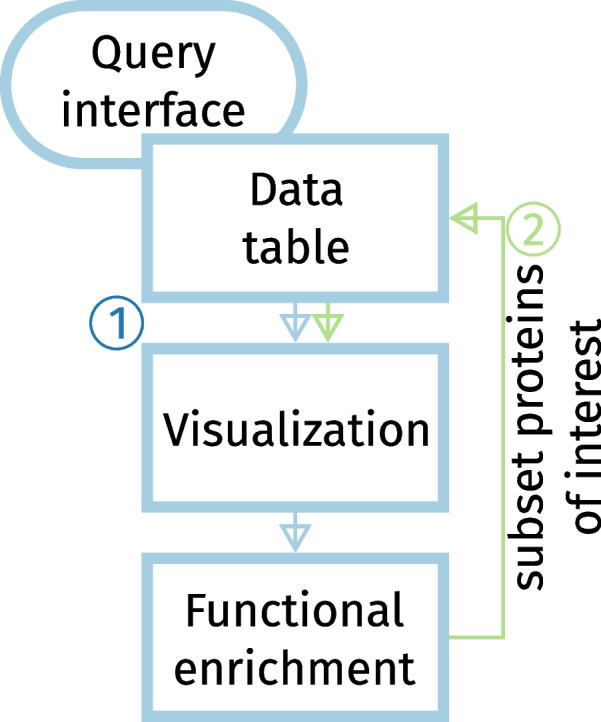
Workflow in the “Differential abundance” tab. Users can define thresholds and select pairwise group comparisons in the query interface, resulting in a data table of differentially abundant proteins. amica’s rich set of plots can be systematically applied to this selection. The data table can be further subsetted using the gene names of over-represented functional terms, allowing users to utilze amica’s visualizations for proteins of interest

amica is a versatile software tool for the analysis, visualization, and interpretation of MS-based proteomics data. amica’s user-friendly interface provides a customizable data analysis workflow, and the results of the analysis can be conveniently exported, shared and reloaded into the amica environment for re-inspection at a later time. The data analysis workflow in amica includes quality control and standard differential expression testing, as well as the integration of PPI networks and pathway and gene ontology enrichment analysis for differentially abundant proteins. This latter feature allows the identification of process-relevant entities that can be used to subset the data table in an iterative manner (Fig. 5). The combination of all these features in one single application will help researchers to focus on, and interpret the biology underlying the results of their proteomics experiment. The code for the application and online documentation can be found at https://github.com/tbaccata/amica and the software is available at https://bioapps.maxperutzlabs.ac.at/app/amica.

## Availability and requirements

**Project name:** amica.

**Project home page:** https://bioapps.maxperutzlabs.ac.at/app/amica

**Operating system(s):** Platform independent.

**Programming language:** R.

**Other requirements:** none for the webserver, R version 4 or higher for local installation.

**License:** GNU GPL version 3.0.

**Any restrictions to use by non-academics:** none.

amica’s source code, online documentation and a formatted example data set are available on https://github.com/tbaccata/amica.

## Supplementary Information

**Additional file 1:** **Supplementary Material — File 1.** Link to a user manual demonstrating every available function and output graphics, as well as extensive tutorials.

## Data Availability

The example dataset was downloaded from the ProteomeXchange Consortium (http://www.proteomexchange.org/) via the PRIDE partner repository with the dataset accession number: PXD016455.

## Abbreviations

MS: Mass spectrometry
PPI: Protein-protein interaction
MS/MS: Tandem-mass spectrum
LFQ: Label-free quantification
VSN: Variance Stabilization Normalization
QC: Quality Control
iBAQ: Intensity-based absolute quantification
MBR: Match between runs

## References

[1] Tyanova S, Temu T, Sinitcyn P, Carlson A, Hein MY, Geiger T (2016). The Perseus computational platform for comprehensive analysis of (prote) omics data. Nat Methods..

[2] Choi M, Chang CY, Clough T, Broudy D, Killeen T, MacLean B (2014). MSstats: an R package for statistical analysis of quantitative mass spectrometry-based proteomic experiments. Bioinformatics..

[3] Gatto L, Lilley KS (2012). MSnbase-an R/Bioconductor package for isobaric tagged mass spectrometry data visualization, processing and quantitation. Bioinformatics..

[4] Tyanova S, Temu T, Cox J (2016). The MaxQuant computational platform for mass spectrometry-based shotgun proteomics. Nat Protoc..

[5] Shah AD, Goode RJ, Huang C, Powell DR, Schittenhelm RB (2019). LFQ-analyst: an easy-to-use interactive web platform to analyze and visualize label-free proteomics data preprocessed with MaxQuant. J Proteome Res..

[6] Kraus M, Mathew Stephen M, Schapranow MP (2020). Eatomics: Shiny exploration of quantitative proteomics data. J Proteome Res..

[7] Gallant JL, Heunis T, Sampson SL, Bitter W (2020). ProVision: a web-based platform for rapid analysis of proteomics data processed by MaxQuant. Bioinformatics..

[8] Teo GC, Polasky DA, Yu F, Nesvizhskii AI (2020). Fast Deisotoping Algorithm and Its Implementation in the MSFragger Search Engine. J Proteome Res..

[9] da Veiga Leprevost F, Haynes SE, Avtonomov DM, Chang HY, Shanmugam AK, Mellacheruvu D (2020). Philosopher: a versatile toolkit for shotgun proteomics data analysis. Nat Methods..

[10] Ritchie ME, Phipson B, Wu D, Hu Y, Law CW, Shi W (2015). limma powers differential expression analyses for RNA-sequencing and microarray studies. Nucleic Acids Res..

[11] Zhu Y, Orre LM, Tran YZ, Mermelekas G, Johansson HJ, Malyutina A (2020). DEqMS: a method for accurate variance estimation in differential protein expression analysis. Mol Cell Proteom..

[12] Teakel SL, Ludescher M, Thejer BM, Poschmann G, Forwood JK, Neubauer H (2020). Protein complexes including PGRMC1 and actin-associated proteins are disrupted by AG-205. Biochem Biophys Res Commun..

[13] Cox J, Hein MY, Luber CA, Paron I, Nagaraj N, Mann M (2014). Accurate proteome-wide label-free quantification by delayed normalization and maximal peptide ratio extraction, termed MaxLFQ. Mol Cell Proteom..

[14] Kong AT, Leprevost FV, Avtonomov DM, Mellacheruvu D, Nesvizhskii AI (2017). MSFragger: ultrafast and comprehensive peptide identification in mass spectrometry-based proteomics. Nat Methods..

[15] Yu F, Haynes SE, Nesvizhskii AI. IonQuant enables accurate and sensitive label-free quantification with FDR-controlled match-between-runs. Mol Cell Proteom. 2021;20:100077. 10.1016/j.mcpro.2021.100077 PMC8131922 33813065

[16] MacCoss MJ, Noble WS, Käll L (2016). Fast and accurate protein false discovery rates on large-scale proteomics data sets with percolator 3.0. J Am Soc Mass Spectrom.

[17] Neuwirth E. RColorBrewer: ColorBrewer palettes; 2014. Available: https://cran.r-project.org/package=RColorBrewer. Accessed 05 Dec 2022.

[18] Conway JR, Lex A, Gehlenborg N (2017). UpSetR: an R package for the visualization of intersecting sets and their properties. Bioinformatics.

[19] Galili T, O’Callaghan A, Sidi J, Sievert C (2018). heatmaply: an R package for creating interactive cluster heatmaps for online publishing. Bioinformatics..

[20] Orchard S, Ammari M, Aranda B, Breuza L, Briganti L, Broackes-Carter F (2014). The MIntAct project–IntAct as a common curation platform for 11 molecular interaction databases. Nucleic Acids Res..

[21] Go CD, Knight JD, Rajasekharan A, Rathod B, Hesketh GG, Abe KT (2021). A proximity-dependent biotinylation map of a human cell. Nature..

[22] Shannon P, Markiel A, Ozier O, Baliga NS, Wang JT, Ramage D (2003). Cytoscape: a software environment for integrated models of biomolecular interaction networks. Genome Res..

[23] Raudvere U, Kolberg L, Kuzmin I, Arak T, Adler P, Peterson H (2019). g: Profiler: a web server for functional enrichment analysis and conversions of gene lists (2019 update). Nucleic Acids Res..

